# Andexanet alpha-induced heparin resistance treated by nafamostat mesylate in a patient undergoing total aortic arch repair for Stanford type A acute aortic dissection: a case report

**DOI:** 10.1186/s40981-024-00690-8

**Published:** 2024-01-29

**Authors:** Yasuhito Suzuki, Mutsuhito Kikura, Shingo Kawashima, Tetsuro Kimura, Yoshiki Nakajima

**Affiliations:** 1 Department of Anesthesiology and Intensive Care, Hamamatsu University School of Medicine, Shizuoka, Japan; 2 Department of Anesthesiology, Hamamatsu Rosai Hospital, Japan Organization of Occupational Health and Safety, Shizuoka, Japan

**Keywords:** Andexanet alpha, Oral Xa inhibitor, Direct oral anticoagulants, Heparin resistance, Nafamostat mesylate, Antithrombin, Cardiovascular surgery

## Abstract

**Background:**

Andexanet alfa, an anti-Xa inhibitor antagonist, induces heparin resistance. Here, we report a case of successful management of cardiopulmonary bypass with andexanet alfa-induced heparin resistance using nafamostat mesylate.

**Case presentation:**

An 84-year-old female, with Stanford type A acute aortic dissection, underwent an emergency surgery for total aortic arch replacement. Andexanet alfa 400 mg was administered preoperatively to antagonize edoxaban, an oral Xa inhibitor. Heparin 300 IU/kg was administered before cardiopulmonary bypass, and the activated clotting time (ACT) was 291 s. The ACT was 361 s after another administration of heparin 200 IU/kg. According to our routine therapy for heparin resistance, an initial dose of nafamostat mesylate 10 mg was administered intravenously, followed by a continuous infusion of 20–30 mg/h. The ACT was prolonged to 500 s, and cardiopulmonary bypass was successfully established thereafter.

**Conclusions:**

This case report presents the successful management of cardiopulmonary bypass with andexanet alfa-induced heparin resistance using nafamostat mesilate. This report presents the successful management of cardiopulmonary bypass with andexanet alfa-induced heparin resistance using nafamostat mesilate.

## Background

Andexanet alfa, an anti-Xa inhibitor antagonist, is a genetically modified decoy protein of human Xa factor that inhibits anticoagulation by reversibly binding to anti-Xa inhibitors [1]. Andexanet alfa possibly causes heparin resistance, preventing the initiation of cardiopulmonary bypass (CPB) [2]. The current 2021 American Society of Cardiovascular Anesthesiologists (SCA) guidelines recommend the use of direct oral anticoagulant (DOAC) antagonists during emergency cardiac surgery in patients receiving DOACs [3]. However, the SCA guidelines do not address the phenomenon of heparin resistance, how to optimally achieve systemic anticoagulation, or how to optimally monitor the coagulation status of patients receiving andexanet alfa [4]. This case report presents the successful management of a CBP with andexanet alfa-induced heparin resistance using nafamostat mesylate, a broad-spectrum synthetic serine protease inhibitor. Moreover, we reviewed the characteristics of patients in previous case reports related to andexanet alpha-induced heparin resistance who underwent cardiovascular surgery.

Written informed consent from the patient and the family for the use of medical records was obtained at hospital admission, and this case report was approved by the Institutional Review Board of the Committee of Ethics at Hamamatsu Rosai Hospital (Hamamatsu, Japan).

## Case presentation

**Fig. 1:**
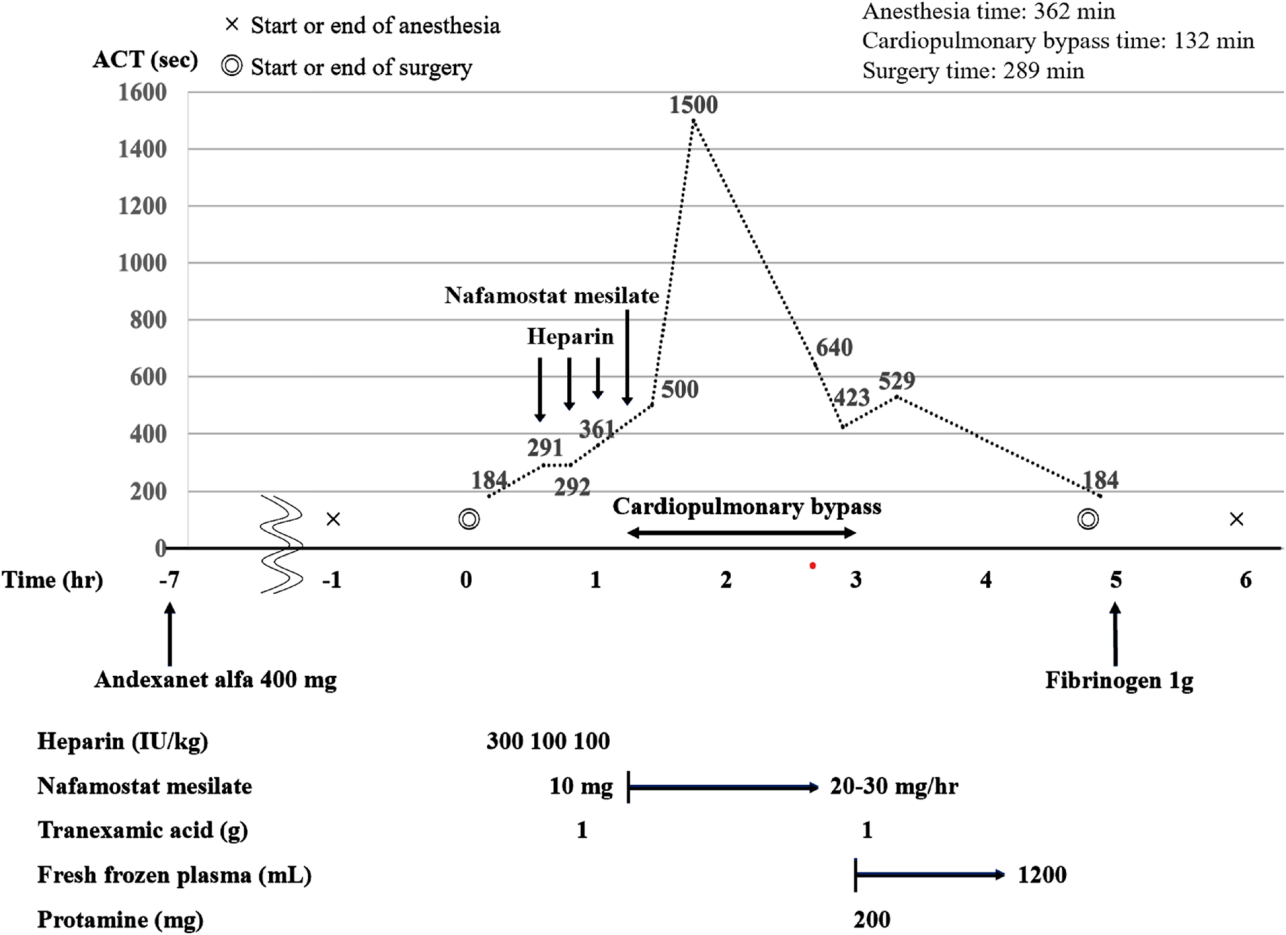
The chronological changes in activated clotting time (ACT) during the perioperative period. Andexanet alfa 400 mg was administered preoperatively. Heparin resistance was observed after administration of 500 IU/kg heparin before cardiopulmonary bypass. ACT was prolonged to 500 s after the administration of an initial dose of nafamostat mesilate 10 mg, followed by a continuous infusion of 20–30 mg/h. Cardiopulmonary bypass was successfully established thereafter ACT, activated clotting time

An 84-year-old female, with Stanford type A acute aortic dissection, underwent emergency surgery for total aortic arch replacement. Owing to a history of cerebral infarction, she was administered edoxaban (an oral Xa inhibitor, DOAC) as maintenance therapy. Preoperative blood examination displayed that the antithrombin III level was within the normal range, but fibrinogen degradation products (FDP) and D-dimer levels were abnormally elevated (Table 1). The changes in the activated clotting time (ACT) are summarized in Fig. 1. The ACT was 184 s immediately after starting surgery, 291 and 361 s after the administration of 300 IU/kg and an additional 200 IU/kg of heparin, respectively (Fig. 1). To antagonize edoxaban, 400 mg of andexanet alfa was administered 6 h before the surgery. Heparin 300 IU/kg was administered before CPB, and the ACT at that time was 291 s. After repeated doses of heparin (200 IU/kg), ACT increased to 361 s. Following our routine therapy for heparin resistance, an initial dose of 10 mg of nafamostat mesylate was intravenously administered, followed by a continuous infusion of the drug at a dose of 20–30 mg/h. The ACT increased to 500 s, and the CPB was successfully established thereafter. After completion of CPB, continuous infusion of nafamostat mesylate was stopped, and protamine 200 mg was administered to reverse the heparin. The ACT decreased to 184 s, and no abnormal bleeding was observed in the surgical field thereafter. In the intensive care unit, only a minimal amount of bleeding was observed from the chest tube drainage. The patient was extubated on the first postoperative day and discharged without adverse events.

**Table 1:** Laboratory blood tests before and after surgery

		Before surgery	After surgery
White blood cell	(/μL)	3900	4000
Hemoglobin	(g/dL)	8.6	8.7
Hematocrit	(%)	26.6	26.2
Platelet	(× 10^4^/μL)	15.8	4.6
PT-INR		1.31	1.64
APTT	(sec)	36.9	38.5
Fibrinogen	(mg/dL)	273	136
FDP	(μg/mL)	23.6 ↑	104.3 ↑
D-dimer	(μg/mL)	6.2 ↑	12.5 ↑
Antithrombin III	(%)	91	–
AST (GOT)	(U/mL)	58	39
ALT (GPT)	(U/mL)	39	25
Blood urea nitrogen	(mg/dL)	19	17
Creatinine	(mg/dL)	1.04	0.95
Creatine kinase	(U/mL)	84	61
C-reactive protein	(mg/dL)	0.06	0.45

*ALT* alanine aminotransferase, *APTT* activated partial thromboplastin time, *AST* aspartate aminotransferase, *FDP* fibrinogen degradation products, *GOT* glutamate oxaloacetate transaminase, *GPT* glutamic pyruvic transaminase, *PT-INR* prothrombin time-international normalized ratio

## Discussion

Andexanet alfa rapidly antagonizes anti-Xa factor inhibitors such as DOACs. Additionally, Andexanet alfa also inhibits the anticoagulant effect of heparin by reversibly binding to the heparin-antithrombin III complex in vitro, resulting in heparin resistance [5, 6] and leading to possible antithrombin III deficiency. This association is supported by the reports that demonstrate that antithrombin administration was effective against heparin resistance induced by andexanet alfa [7–9].

Nafamostat mesilate, the drug of choice in the present case report, directly inhibits a variety of proteolytic enzymes, including VIIa, Xa, thrombin, kallikrein, platelet aggregation, plasmin, complement, and trypsin [10]. Nafamostat mesylate exerts anticoagulant effects by acting on several pathways regardless of antithrombin III. Furthermore, nafamostat mesylate effectively and safely treats heparin resistance without increasing the risk of ischemic stroke or myocardial infarction in patients undergoing cardiovascular surgery [11]. Additionally, we considered that ACT could be used as an appropriate coagulation monitor in patients with andexanet alfa-induced heparin resistance who are treated with nafamostat mesylate, although further studies are needed to verify the optimal monitoring of the coagulation status of patients receiving andexanet alfa. Therefore, nafamostat mesylate is an effective alternative anticoagulant therapy when antithrombin is ineffective or unavailable for heparin resistance induced by andexanet alfa.

To characterize heparin resistance induced by andexanet alfa among patients who underwent cardiovascular surgery with CPB, we reviewed case reports from PubMed, MEDLINE, and J Dream III. Additionally, the following search methods were used to identify cases. We searched PubMed, Medline, and J Dream from their inception until September 31, 2023. Subject headings and search field tags for titles, abstracts, and keywords were used to facilitate the search. The following terms were used to search for relevant records: “andexanet alfa,” “heparin resistance,” “cardiopulmonary bypass,” and “cardiovascular surgery.” We finally detected nine cases of heparin resistance due to andexanet alfa, and those nine cases (in eight articles) were published as journal articles [7, 9, 12–17]. Table 2 summarizes the characteristics of andexanet alpha-induced heparin resistance in 10 cases, including nine cases from the previous reports described above and one case from the present case report. Table 3 summarizes previous case reports of andexanet alpha-induced heparin resistance. As presented in Tables  2 and 3, 100.0% of the patients underwent emergency surgery for acute aortic dissection. Andexanet alfa was administered preoperatively in 90.0% of all cases. To treat andexanet alfa-induced heparin resistance, the inefficacy of an additional dose of heparin was 90.0%, the inefficacy of fresh frozen plasma was 100%, and that of antithrombin was 25.0%. Only nafamostat mesylate was effective in all patients. Furthermore, in a case report (Tables 2 and 3), andexanet alfa-induced heparin resistance was successfully treated with nafamostat mesilate after antithrombin failed to prolong the ACT [17].

**Table 2:** Characteristics of andexanet alpha-induced heparin resistance

Total, *n*	10 cases
Age (year) median (range)	73.5 (46–87)
Sex, male; *n* (%)	5 (50.0)
Body weight (kg) median (range)	76 (50–120)
Direct oral anticoagulants	
Apixaban, *n* (%)	5 (50.0)
Edoxaban, *n* (%)	3 (30.0)
Rivaroxaban,* n* (%)	2 (20.0)
Surgical diagnosis	
Stanford type A acute aortic dissection, *n* (%)	7 (70.0)
Ruptured abdominal aortic aneurysm, *n* (%)	2 (20.0)
Left ventricular free wall rupture, *n* (%)	1 (10.0)
Emergency surgery, *n* (%)	10 (100.0)
Andexanet alfa administration	
Before surgery	9 (90.0)
During cardiopulmonary bypass	1 (10.0)
Dose of andexanet alfa (mg)	
800 mg IV + 8 mg/min div	1 (10.0)
400 mg IV + 4 mg/min div	8 (80.0)
Treatment for heparin resistance	
Heparin addition, *n* (%)	10 (100.0)
Inefficacy of heparin addition, *n* (%)	9 cases in 10 cases (90.0)
Fresh frozen plasma, *n* (%)	2 (20.0)
Inefficacy of fresh frozen plasma, *n* (%)	2 cases in 2 cases (100.0)
Antithrombin, *n* (%)	4 (40.0)
Inefficacy of antithrombin, n (%)	1 case in 4 cases (25.0)
Nafamostat mesilate,* n* (%)	2 (20.0)
Inefficacy of nafamostat mesilate, *n* (%)	0 case in 2 cases (0.0)
Complication of thrombus, *n* (%)	3 (30.0)

*IV* Intravenous

**Table 3:** Summary of the previous case reports with andexanet alpha-induced heparin resistance

Disease	Age	Sex	BW (kg)	Direct oral anticoagulants	Andexanet alfa administration	Dose of andexanet alfa	Treatment for heparin resistance	Reference
Type A aortic dissection	81	Female	53	Edoxaban	Before surgery	400 mg + 4 mg/min	Additional heparin 3000 IU, antithrombin	Honda J, et al. [9]
Type A aortic dissection	67	Male	80	Apixaban	During cardiopulmonary bypass	800 mg + 8 mg/min	Additional heparin, antithrombin	Brenner B, et al. [13]
Type A aortic dissection	76	Female	67	Apixaban	Before surgery	400 mg + 4 mg/min	Additional heparin, FFP	Brenner B, et al. [13]
Type A aortic dissection	75	Male	76	Edoxaban	Before surgery	400 mg + 4 mg/min	Additional heparin	Al-Attar N, et al. [14]
Type A aortic dissection	46	Male	120	Apixaban	Before surgery	400 mg + 4 mg/min	Additional heparin	Flaherty D, et al. [15]
Type A aortic dissection	87	Female	65	Apixaban	Before surgery	Non-described	Additional heparin, antithrombin, nafamostat mesylate	Kitaura A, et al. [17]
Ruptured abdominal aortic aneurysm	70	Male	84	Rivaroxaban	Before surgery	400 mg + 4 mg/min	Additional heparin, FFP	Eche IM, et al. [12]
Ruptured abdominal aortic aneurysm	70	Male	85	Rivaroxaban	Before surgery	400 mg + 4 mg/min	Additional heparin, FFP	Watson CJ, et al. [16]
Left ventricular free wall rupture	72	Male	Non described	Apixaban	Before surgery	400 mg + 4 mg/min	Additional heparin, 1000 IU antithrombin	Apostel HJCL, et al. [7]

Previous case reports indicated that andexanet alfa is effective for hemostasis in cardiovascular surgery with CPB [14, 18]. However, as summarized in Tables 2 and 3, thrombus formation during CPB was noted in three patients (30.0%). Therefore, andexanet alfa should be cautiously administered in patients undergoing cardiovascular surgery when using CPB.

In acute aortic dissection, the dissection of the tunica media in the aortic wall results in the release of tissue factor from the false lumen, triggering an enhanced activation of the coagulation system, resulting in high levels of FDP and D-dimer [19]. Indeed, the FDP and D-dimer levels were abnormally increased in this case report, as presented in Table 1. Furthermore, heparin resistance is known to occur during aortic dissection regardless of antithrombin III levels [20]. As summarized in Tables  2 and 3, the ineffectiveness of antithrombin in addressing heparin resistance due to andexanet alfa was 25.0%. This suggests that andexanet alfa-induced heparin resistance is not fully attributable to antithrombin III deficiency. Therefore, considering the literature review of previous case reports and the perioperative characteristics of the present case report, we propose that heparin resistance due to andexanet alfa might be related to preoperative hypercoagulability in patients with acute aortic dissection. Nafamostat mesilate is an effective agent for an anti-hypercoagulable state after administration of andexanet alfa to safely initiate CPB due to its anticoagulant effect regardless of antithrombin III and a short life of 8 min. Also, Further studies are needed regarding the dosage of nafamostat mesylate in heparin-resistant patients with andexanet alfa. In this case, an initial dose of 10 mg of nafamostat mesylate was administered followed by a continuous infusion of 20–30 mg/h. After administration, the ACT increased to 500 s and CPB was successfully established. This dosage is approximately the same as the recommended dosage of nafamostat mesilate listed in the attached document.

In conclusion, nafamostat mesylate is an effective anticoagulant therapy for heparin resistance induced by andexanet alfa, thereby allowing the safe initiation of CPB.

## Data Availability

Data sharing does not apply to this article because no datasets were generated or analyzed in the current case report.

## Abbreviations

ACT: Activated clotting time
CPB: Cardiopulmonary bypass
DOACs: Direct oral anticoagulants
FDP: Fibrinogen degradation product
SCA: Society of Cardiovascular Anesthesiologists
